# Real-world safety profile of mitotane in adrenocortical carcinoma: a retrospective cross-sectional study integrating pharmacovigilance and interpretable machine learning

**DOI:** 10.3389/fendo.2026.1862262

**Published:** 2026-07-22

**Authors:** Jun Wang, Yang Yang, Ke Xu, Ning Hou, Chenyu Guo

**Affiliations:** 1 Department of Pharmacy, Shandong Provincial Hospital Affiliated to Shandong First Medical University, Jinan, Shandong, China; 2 Department of Pharmacy, Yantai Yuhuangding Hospital Affiliated to Qingdao University, Yantai, Shandong, China; 3 Department of Reproductive Medicine, Shandong Provincial Hospital Affiliated to Shandong First Medical University, Jinan, Shandong, China; 4 Department of Obstetrics and Gynecology, Shandong Provincial Hospital Affiliated to Shandong First Medical University, Jinan, Shandong, China; 5 State Key Laboratory of Neurology and Oncology Drug Development, Nanjing, Jiangsu, China

**Keywords:** clinical prioritization, cross-sectional study, disproportionality analysis, FAERS, machine learning, mitotane

## Abstract

**Background:**

Mitotane remains the cornerstone of adrenocortical carcinoma. However, its real-world safety profile is poorly defined. This study systemically evaluated mitotane-associated adverse events (AEs) and characterized multivariable patterns of serious outcomes using a combined pharmacovigilance and machine learning approach.

**Methods:**

Mitotane-related AE reports (2004-2025) were extracted from the FDA Adverse Event Reporting System (FAERS). Disproportionality analyses were performed via four algorithms. Detected signals were categorized using a clinical priority scoring system based on signal strength, mortality, seriousness, and existing evidence. Time-to-onset was evaluated utilizing Kaplan-Meier methods. Elastic net logistic regression and Extreme Gradient Boosting (XGBoost) models characterized features associated with serious outcomes, with performance assessed by the area under the receiver operating characteristic curve (AUROC).

**Results:**

Among 870 identified reports, 45.7% involved serious outcomes. We detected 75 safety signals, predominantly involving gastrointestinal, neurological, endocrine, and metabolic systems. Notably, 21 signals (28.0%) were assigned moderate clinical priority, with adrenal insufficiency as the highest-priority signal. Time-to-onset analysis showed that endocrine AEs had a significantly longer onset time than non-endocrine AEs. Both models demonstrated consistent characterization of multivariable patterns associated with serious outcomes (elastic net AUROC = 0.830, XGBoost AUROC = 0.869). Key contributors to serious outcomes included reporting country, medical event designation, organ system involvement, and medication complexity.

**Conclusion:**

This large-scale study provides a real-world safety assessment of mitotane. Integrating signal detection, clinical prioritization, and interpretable machine learning reveals multivariable patterns underlying serious AEs. These findings support a risk-stratified monitoring approach to enhance the clinical safety of mitotane therapy.

## Introduction

Adrenocortical carcinoma (ACC) is a rare and highly aggressive malignancy originating from the adrenal cortex. According to data from the National Cancer Institute’s Surveillance, Epidemiology and End Results database, the age-adjusted annual incidence of ACC in the United States ranges from 0.72 to 1.02 per million (1, 2). The disease exhibits a bimodal age distribution, with incidence peaks in both the first and fourth decades of life (3) and demonstrates a slight female predominance (4). Clinically, approximately 60% of ACC patients present with hormone excess syndromes, including Cushing’s syndrome, virilization, or feminization (5). Despite advancements in treatment, the prognosis of ACC remains poor, with a median overall survival of 3.21 years and a 5-year survival rate ranging from 15% to 44% (6).

**Figure 1:**
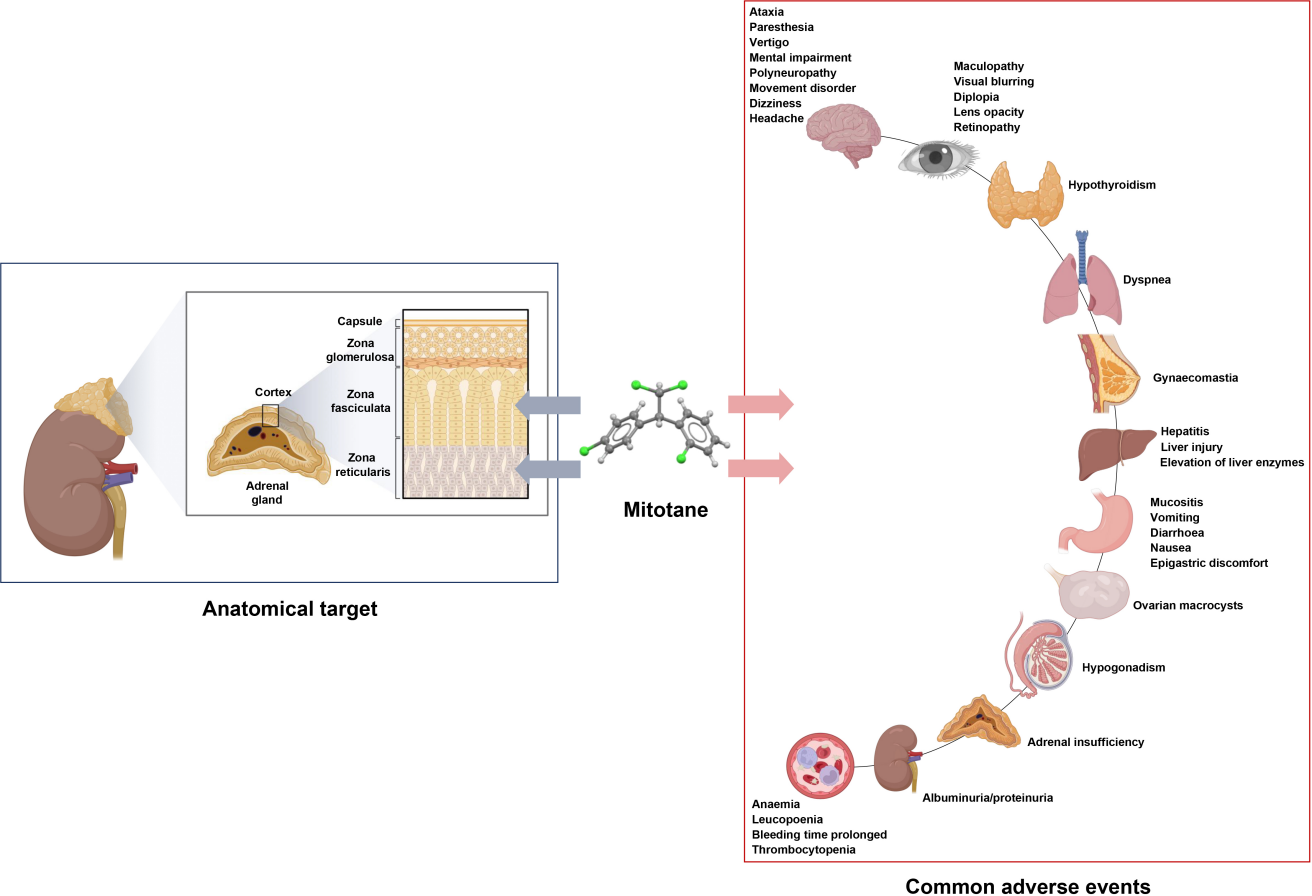
Schematic overview of mitotane’s anatomical target and its adverse event profile. The left panel depicts the anatomical and histological structure of the adrenal cortex, the primary target where mitotane exerts its selective cytotoxic effects. The right panel provides a comprehensive mapping of common adverse events associated with mitotane.

Radical surgery remains the only potentially curative treatment for localized ACC (7). However, many cases are diagnosed at advanced stages, where surgical resection is often unfeasible. Even in patients who underwent complete surgical resection, the risk of recurrence remains high, with local recurrence rates ranging from 19% to 34% (8). Owing to the aggressive biology of ACC and its high risk of recurrence, effective therapeutic options beyond surgery remain limited. Mitotane (o, p’-DDD), a derivative of dichlorodiphenyltrichloroethane, is the only pharmacological agent specifically approved by the U.S. Food and Drug Administration (FDA) and the European Medicines Agency for ACC treatment (9). Mitotane both exerts cytotoxic effects and suppresses steroidogenesis, selectively targeting adrenocortical cells (10) (**Figure 1**). Retrospective studies suggest that adjuvant mitotane therapy following surgery may improve disease-free survival and overall survival (11–13). Consequently, clinical guidelines recommend mitotane for patients at high risk of recurrence post-surgery (14–16).

Despite its therapeutic benefits, mitotane is associated with significant toxicities, primarily affecting the gastrointestinal system, nervous system, metabolism, and endocrine functions (17) (**Figure 1**). However, current safety data are predominantly derived from retrospective studies (17, 18) and small-scale, single-arm clinical trials, with only a limited number of phase III investigations available (19, 20). Given the rarity of ACC, these studies commonly suffer from small sample sizes and limited ethnic diversity, which may constrain the generalizability of their findings. Moreover, comprehensive population-based pharmacovigilance assessments remain scarce, leaving gaps in the understanding of the real-world safety profile of mitotane. Notably, existing evidence is primarily restricted to frequency-based descriptions, often overlooking clinical seriousness, outcomes, and temporal patterns. Furthermore, it lacks a systematic characterization of the multivariable factors associated with serious AEs.

To address these gaps, we conducted a post-marketing pharmacovigilance analysis of mitotane via the FDA Adverse Event Reporting System (FAERS). In addition to conventional disproportionality-based signal detection, clinical priority analyses were employed to integrate clinical seriousness and outcomes into signal interpretation. Furthermore, machine learning approaches were implemented as complementary tools to characterize multivariable patterns associated with serious outcomes under both linear and nonlinear assumptions. The objective was not to develop or validate a clinical prediction model, but rather to provide complementary insights into factors associated with serious reports within the FAERS database.

## Materials and methods

### Study design

This is a retrospective, cross-sectional pharmacovigilance study, which was performed in accordance with the Declaration of Helsinki.

### Data source

The data used in this study were obtained from the FAERS, a publicly available pharmacovigilance database designed for post-marketing surveillance of drugs and therapeutic biologics. FAERS is among the largest spontaneous reporting systems globally, comprising over 20 million reports of AEs, medication errors, and product quality complaints voluntarily submitted by healthcare professionals, consumers, and manufacturers to the FDA. The FAERS dataset comprises seven data files, including demographic and administrative information (DEMO), drug information (DRUG), adverse drug reaction information (REAC), patient outcome information (OUTC), information on report sources (RPSR), drug therapy start dates and end dates (THER), and indications for use/diagnosis (INDI).

### Data processing procedure

**Figure 2:**
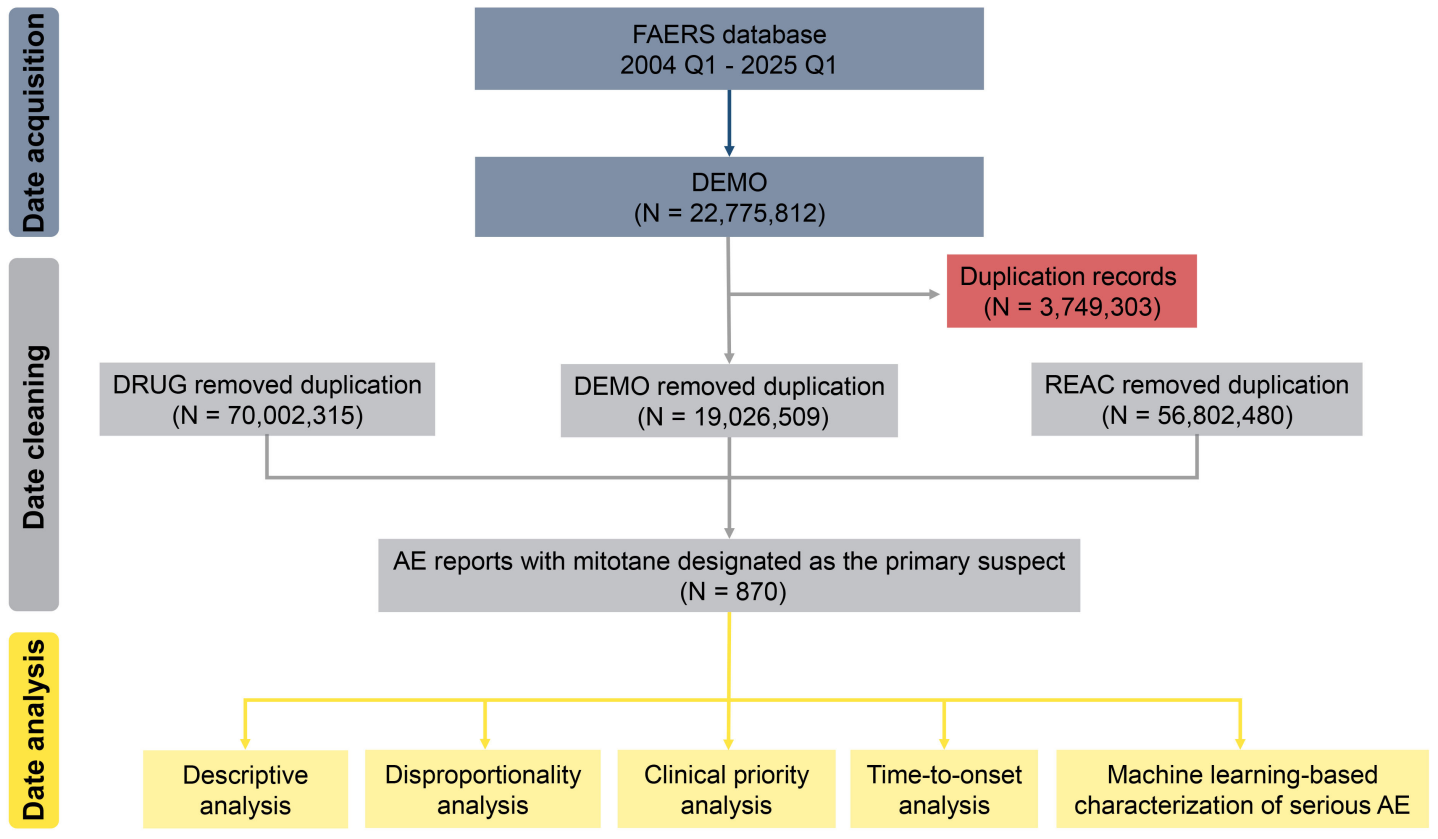
Flowchart of mitotane-related adverse event report selection from the FAERS database.

In this study, mitotane was designated as the drug of interest. To ensure exhaustive data retrieval, we also identified AE reports by using diverse names of mitotane such as “ortho, para-DDD”, “ortho, para DDD”, “o, p-DDD”, “Mytotan”, “Lysodren”, “Chlodithane”, “Chloditan”, “Khloditan”, referencing the MeSH dataset from NCBI. The analysis period covered Q1–2004 to Q1 2025, corresponding to the most recent FAERS update available at the time of data extraction. Inclusion was restricted to reports in which mitotane was explicitly listed as the primary suspect drug. AEs were categorized according to MedDRA (version 27.1) at both the System Organ Class (SOC) and the Preferred Term (PT) levels. If a PT was associated with multiple SOCs, the primary SOC as designated by MedDRA was selected. The data handling procedures strictly adhered to FDA guidelines. The data selection and analysis process is illustrated in **Figure 2**.

### Statistical analysis

Descriptive statistics were employed to summarize the distributions of sex, age, weight, reporting country, reporter type, indication, and outcomes in the AE reports. Differences in the distribution of report-level characteristics between the serious and non-serious outcome groups were descriptively assessed via the chi-square test or Fisher’s exact test, as appropriate. To detect potential safety signals associated with mitotane, disproportionality analyses were performed using four complementary algorithms, including the reporting odds ratio (ROR) (21–23), proportional reporting ratio (PRR) (24), multi-item Gamma Poisson Shrinker (MGPS)-based empirical Bayesian geometric mean (EBGM) (25), and Bayesian confidence propagation neural network (BCPNN) (26, 27). A safety signal was considered positive only when the predefined criteria of all four algorithms were met simultaneously. Detailed definitions and thresholds for these algorithms are provided in **Supplementary Table 1**. To control for multiple testing across PTs, the Benjamini-Hochberg false discovery rate procedure was applied to adjust *P* values derived from disproportionality analysis, and q values were reported as adjusted measures of statistical significance.

A semiquantitative clinical priority scoring system was subsequently applied to rank identified safety signals based on five complementary dimensions derived from pharmacovigilance and regulatory science literature (28, 29) (1): the number of target AE reports (2), the lower limit of the 95% CI for the ROR (3), mortality proportion (4), classification as designated medical events (DMEs) or important medical events (IMEs), and (5) evidence assessment. Importantly, DME/IME classification reflects the intrinsic clinical importance of MedDRA PTs and is derived from predefined regulatory pharmacovigilance lists established by the European Medicines Agency (30, 31), rather than from individual FAERS case-level seriousness outcomes. Therefore, DME/IME status is independent of FAERS seriousness definitions (e.g., death, hospitalization, disability, or life-threatening condition), which are applied at the report level. Each dimension was independently scored on a three-level ordinal scale (0-2), and equal weighting was applied to maintain methodological neutrality across heterogeneous evidence types. The composite score was obtained by summing the five dimensions (range 0-10). The detailed scoring criteria for each dimension, including cut-off thresholds and the structured evidence assessment framework (++, +, −), are provided in **Supplementary Table 2**. Thresholds for low (0-4), moderate (5-7), and high (8-10) clinical priority were predefined according to previously published methodologies (28, 29), and were not data-driven. This categorization reflects a qualitative stratification of clinical relevance rather than an empirical optimization procedure. To avoid overlap with FAERS seriousness definitions, variables derived from this scoring system were excluded from machine learning analyses.

In the FAERS database, time to onset (TTO) was defined as the interval between the initiation of drug therapy (START_DT in the THER file) and the occurrence of an AE (EVENT_DT in the DEMO file). The Kaplan-Meier method was applied to characterize the temporal distribution of AEs, and differences between subgroups were assessed using the log-rank test. Box plots with jittered points were employed to visualize the TTO distribution for safety signals with moderate and high clinical priority.

To assess the potential impact of concomitant medications on signal detection, a sensitivity analysis was conducted using a restricted subset of reports in which mitotane was recorded as the sole primary suspect drug. The same analytical procedures applied in the primary analysis were repeated in this monotherapy subset, and the concordance of identified safety signals between the two analyses was evaluated as a measure of signal robustness.

In addition, machine learning analyses were conducted as exploratory extensions of conventional pharmacovigilance, with an emphasis on interpretability rather than prediction. Prior to analytical modeling, missing values in selected variables were addressed using Multiple Imputation by Chained Equations. Age was imputed using Bayesian linear regression, whereas sex and reporter type were imputed using polytomous logistic regression. Five imputed datasets were generated, and a completed dataset was derived from the imputation procedure for downstream analyses. After feature engineering, the processed variables capturing demographic characteristics, reporting-related information, AE profiles, and medication complexity were used in the modeling frameworks. A regularized logistic regression model with elastic net penalization was used to characterize linear associations between covariates and serious outcomes, whereas a gradient boosting decision tree model (Extreme Gradient Boosting, XGBoost) was applied to explore potential nonlinear relationships and interactions. To evaluate the stability of the identified multivariable association patterns across analytical frameworks, the area under the receiver operating characteristic curve (AUROC) was calculated using 10-fold cross-validation. These analyses were intended for exploratory characterization of report seriousness rather than development of a clinical prediction model. Feature importance was characterized via standardized regression coefficients for the logistic regression model and SHapley Additive exPlanations (SHAP) for the XGBoost model, respectively. To evaluate the consistency of the multivariable patterns identified by the two approaches, the estimated probabilities generated by the two models were correlated. In addition, to assess the potential influence of low-frequency AEs on the machine learning analyses, a sensitivity analysis was performed by excluding PT-level features reported in fewer than 10 cases. The machine learning models were subsequently rebuilt using the reduced feature set, and the resulting model performance and feature importance profiles were compared with those from the primary analysis.

All data processing and statistical analyses were conducted using R software (version 4.3.0). All statistical tests were two-sided, with *P* < 0.05 considered statistically significant.

## Results

### Characteristics of mitotane-related AE reports

A total of 19, 026, 509 AE reports were retrieved during the study period (Q1 2004-Q1 2025) after deduplication. Of these, 870 reports identified mitotane as the primary suspect drug. The characteristics of these reports are summarized in **Table 1**. Among the patients, females represented a greater proportion than males (63.1% vs. 32.1%). The median weight was 66 kg, and the 18–64 years age group accounted for 57.6% of all cases. Most reports originated from the United States (56.6%), followed by France (20.0%) and Canada (3.1%). The majority of reports were submitted by consumers (63.6%) and physicians (14.6%). A significant proportion of patients (70.0%) were diagnosed with ACC. Serious AEs were reported in 45.7% of cases, with hospitalization being the most frequently reported serious outcome.

**Table 1:** Characteristics of AE reports related to mitotane in the FAERS database.

Characteristics	Mitotane(N=870)
Sex	
Male	279 (32.1%)
Female	549 (63.1%)
Unknown	42 (4.8%)
Age	
0-17	57 (6.6%)
18-64	501 (57.6%)
≥65	170 (19.5%)
Unknown	142 (16.3%)
Weight	
Median, kg (Q1-Q3)	66 (51-82)
Unknown	757 (87.0%)
Reporting country	
United States	492 (56.6%)
France	174 (20.0%)
Canada	27 (3.1%)
Germany	23 (2.6%)
Colombia	22 (2.5%)
Others	91 (10.5%)
Unknown	41 (4.7%)
Reporter type	
Consumer	553 (63.6%)
Physician	127 (14.6%)
Pharmacist	48 (5.5%)
Health Professional	25 (2.9%)
Unknown	117 (13.4%)
Indications	
Adrenocortical carcinoma	609 (70.0%)
Others	74 (8.5%)
Unknown	187 (21.5%)
Reporting year	
2004	7 (0.8%)
2005	3 (0.3%)
2006	6 (0.7%)
2007	21 (2.4%)
2008	23 (2.6%)
2009	23 (2.6%)
2010	16 (1.8%)
2011	24 (2.8%)
2012	43 (4.9%)
2013	23 (2.6%)
2014	26 (3.0%)
2015	27 (3.1%)
2016	36 (4.1%)
2017	33 (3.8%)
2018	50 (5.7%)
2019	18 (2.1%)
2020	19 (2.2%)
2021	57 (6.6%)
2022	87 (10.0%)
2023	99 (11.4%)
2024	117 (13.4%)
2025Q1	112 (12.9%)
Outcome	
Serious	398 (45.7%)
Non-serous	472 (54.3%)
Serious outcome	
Hospitalization	169 (42.5%)
Death	55 (13.8%)
Life-Threatening	8 (2.0%)
Disability	5 (1.3%)
Congenital Anomaly	1 (0.3%)
Others	160 (40.2%)

### Descriptive comparison of serious and non-serious outcome reports

We compared the distribution of report characteristics between reports with and without serious outcomes (**Table 2**). Significant differences were observed in sex, age, reporting country, and reporter type, whereas patient weight showed a broadly similar distribution. At the PT level (**Supplementary Table 3**), adrenal insufficiency, asthenia, hypothyroidism, and neutropenia were more frequently reported in serious outcome reports, while fatigue, nausea, and diarrhea were more common in non-serious cases. These descriptive differences provided the contextual information for subsequent signal prioritization and machine learning analyses.

**Table 2:** Baseline characteristics of mitotane-related adverse event reports stratified by outcome seriousness.

Characteristics	Serious outcome group (N = 398)	Non-serious outcome group (N = 472)	P value
Sex			
Male	129 (32.4%)	150 (31.8%)	
Female	259 (65.1%)	290 (61.4%)	0.012
Unknown	10 (2.5%)	32 (6.8%)	
Age			
0-17	33 (8.3%)	24 (5.1%)	<0.001
18-64	247 (62.1%)	254 (53.8%)	
≥65	77 (19.3%)	93 (19.7%)	
Unknown	41 (10.3%)	101 (21.4%)	
Weight			
Median, kg (Q1-Q3)	66 (51-82)	68 (43-77)	0.872
Unknown	304 (76.4%)	453 (96.0%)	
Reporting country			
United States	142 (35.7%)	350 (74.2%)	
France	81 (20.4%)	93 (19.7%)	
Canada	27 (6.8%)	0 (0%)	
Germany	21 (5.3%)	2 (0.4%)	<0.001
Colombia	22 (5.5%)	0 (0%)	
Others	73 (18.3%)	18 (3.8%)	
Unknown	32 (8.0%)	9 (1.9%)	
Reporter type			
Consumer	220 (55.3%)	333 (70.6%)	
Physician	78 (19.6%)	49 (10.4%)	
Pharmacist	16 (4.0%)	32 (6.8%)	<0.001
Health Professional	20 (5.0%)	5 (1.1%)	
Unknown	64 (16.1%)	53 (11.2%)	

### Disproportionality analysis and signal detection

In total, 870 reports encompassing 3, 334 AEs and 686 PT-level classifications were analyzed. Using the ROR, PRR, EBGM, and BCPNN algorithms, 75 PT-level safety signals were detected. After Benjamini-Hochberg false discovery rate correction, all 75 identified PT-level signals remained statistically significant (q < 0.05), indicating robustness of the detected disproportionality signals. Detailed results of the disproportionality analysis are shown in **Table 3**. It should be noted that all identified signals refer to MedDRA PTs, which represent standardized AE descriptors in pharmacovigilance and are distinct from FAERS seriousness outcome classifications.

**Table 3:** PT-level safety signals of mitotane in the FAERS database.

PT	N	ROR (95%Cl)	PRR (χ2)	EBGM (EBGM05)	IC (IC025)	P value	q value
Nausea	192	7.21 (6.15-8.47)	5.84 (800.58)	5.84 (4.98)	2.55 (2.28)	<0.001	<0.001
Fatigue	189	7.18 (6.12-8.44)	5.84 (787.38)	5.84 (4.97)	2.55 (2.28)	<0.001	<0.001
Diarrhea	118	4.99 (4.11-6.06)	4.45 (325.67)	4.45 (3.67)	2.15 (1.83)	<0.001	<0.001
Vomiting	95	5.36 (4.33-6.64)	4.89 (300.21)	4.88 (3.95)	2.29 (1.92)	<0.001	<0.001
Off-label use	91	2.83 (2.28-3.52)	2.64 (96.5)	2.64 (2.12)	1.40 (1.06)	<0.001	<0.001
Dizziness	81	4.18 (3.32-5.25)	3.88 (177.5)	3.88 (3.09)	1.96 (1.57)	<0.001	<0.001
Decreased appetite	69	7.29 (5.70-9.32)	6.79 (344.6)	6.79 (5.31)	2.76 (2.29)	<0.001	<0.001
Asthenia	65	4.36 (3.39-5.62)	4.11 (155.92)	4.11 (3.19)	2.04 (1.6)	<0.001	<0.001
Malignant neoplasm progression	54	13.87 (10.53-18.27)	13.07 (604.41)	13.06 (9.92)	3.71 (3.02)	<0.001	<0.001
Adrenal insufficiency	51	116.03 (87.39-154.07)	109.29 (5447.92)	108.75 (81.90)	6.76 (4.73)	<0.001	<0.001
Weight decreased	45	3.99 (2.96-5.39)	3.84 (95.72)	3.84 (2.84)	1.94 (1.42)	<0.001	<0.001
Somnolence	37	4.54 (3.26-6.31)	4.39 (97.65)	4.39 (3.15)	2.13 (1.53)	<0.001	<0.001
Abdominal discomfort	28	4 (2.74-5.83)	3.9 (60.92)	3.90 (2.68)	1.96 (1.28)	<0.001	<0.001
Balance disorder	25	6.97 (4.68-10.38)	6.8 (124.14)	6.80 (4.57)	2.76 (1.9)	<0.001	<0.001
Product use complaint	23	38.25 (25.27-57.9)	37.27 (810.99)	37.21 (24.58)	5.22 (3.29)	<0.001	<0.001
Hypothyroidism	22	17.07 (11.18-26.06)	16.66 (324.09)	16.65 (10.9)	4.06 (2.7)	<0.001	<0.001
Drug interaction	22	3.34 (2.19-5.11)	3.29 (35.23)	3.28 (2.15)	1.72 (0.97)	<0.001	<0.001
Memory impairment	22	3.85 (2.52-5.88)	3.78 (45.19)	3.78 (2.47)	1.92 (1.14)	<0.001	<0.001
Drug level above therapeutic	21	116.81 (75.68-180.29)	114.01 (2340.72)	113.43 (73.48)	6.83 (3.59)	<0.001	<0.001
Disturbance in attention	20	8.77 (5.63-13.67)	8.59 (134.5)	8.59 (5.51)	3.1 (2.02)	<0.001	<0.001
Adverse event	19	4.99 (3.17-7.86)	4.9 (59.24)	4.9 (3.11)	2.29 (1.38)	<0.001	<0.001
Gamma-glutamyl transferase increased	19	20.17 (12.80-31.79)	19.75 (338.32)	19.73 (12.52)	4.3 (2.7)	<0.001	<0.001
Neutropenia	18	3.26 (2.04-5.19)	3.21 (27.55)	3.21 (2.01)	1.68 (0.85)	<0.001	<0.001
Hyponatremia	18	7.72 (4.84-12.31)	7.58 (103.03)	7.58 (4.75)	2.92 (1.82)	<0.001	<0.001
Accidental exposure to product	17	4.37 (2.70-7.06)	4.30 (43.23)	4.30 (2.66)	2.1 (1.17)	<0.001	<0.001
Hypercholesterolemia	17	45.18 (27.94-73.06)	44.32 (718.7)	44.23 (27.36)	5.47 (3.01)	<0.001	<0.001
Vertigo	15	5.90 (3.54-9.84)	5.82 (60.03)	5.82 (3.49)	2.54 (1.43)	<0.001	<0.001
Hepatic enzyme increased	14	5.06 (2.99-8.59)	5 (44.93)	5 (2.95)	2.32 (1.23)	<0.001	<0.001
Dysgeusia	14	4.39 (2.59-7.44)	4.33 (36.04)	4.33 (2.56)	2.12 (1.07)	<0.001	<0.001
Febrile neutropenia	13	4.81 (2.78-8.32)	4.75 (38.62)	4.75 (2.75)	2.25 (1.13)	<0.001	<0.001
Adrenocortical insufficiency acute	13	181.38 (104.66-314.35)	178.69 (2278.57)	177.25 (102.27)	7.47 (2.92)	<0.001	<0.001
Brain fog	13	33.65 (19.45-58.22)	33.16 (405.11)	33.12 (19.14)	5.05 (2.55)	<0.001	<0.001
Cognitive disorder	11	5.71 (3.15-10.35)	5.65 (42.19)	5.65 (3.12)	2.5 (1.19)	<0.001	<0.001
Alanine aminotransferase increased	10	3.86 (2.07-7.20)	3.83 (20.95)	3.83 (2.05)	1.94 (0.73)	<0.001	<0.001
Disorientation	10	5.92 (3.17-11.04)	5.86 (40.41)	5.86 (3.14)	2.55 (1.15)	<0.001	<0.001
Neurotoxicity	9	13.05 (6.76-25.17)	12.92 (99.03)	12.92 (6.70)	3.69 (1.64)	<0.001	<0.001
Ataxia	9	17.92 (9.29-34.57)	17.75 (142.19)	17.73 (9.19)	4.15 (1.81)	<0.001	<0.001
Hyperkalemia	8	5.54 (2.76-11.11)	5.5 (29.48)	5.5 (2.74)	2.46 (0.91)	<0.001	<0.001
Ovarian cyst	8	18.55 (9.24-37.22)	18.39 (131.50)	18.37 (9.16)	4.20 (1.68)	<0.001	<0.001
Hypogonadism	7	163.47 (77.48-344.87)	162.16 (1112.97)	160.97 (76.3)	7.33 (1.91)	<0.001	<0.001
Gastrointestinal toxicity	7	39.04 (18.54-82.19)	38.73 (256.92)	38.67 (18.37)	5.27 (1.73)	<0.001	<0.001
Hepatocellular injury	7	9.82 (4.67-20.66)	9.75 (54.97)	9.74 (4.63)	3.28 (1.19)	<0.001	<0.001
Electrocardiogram QT prolonged	7	4.62 (2.20-9.72)	4.59 (19.69)	4.59 (2.18)	2.2 (0.64)	0.001	0.001
Hypertriglyceridemia	7	28.77 (13.67-60.56)	28.55 (185.9)	28.51 (13.55)	4.83 (1.66)	<0.001	<0.001
Encephalopathy	7	6.93 (3.29-14.57)	6.88 (35.20)	6.88 (3.27)	2.78 (0.96)	<0.001	<0.001
Precocious puberty	6	248.81 (110.97-557.87)	247.10 (1454.24)	244.35 (108.98)	7.93 (1.67)	<0.001	<0.001
Liver function test abnormal	6	4.74 (2.12-10.57)	4.71 (17.56)	4.71 (2.11)	2.24 (0.53)	0.002	0.002
Blood alkaline phosphatase increased	6	5.59 (2.5-12.47)	5.56 (22.44)	5.55 (2.49)	2.47 (0.65)	0.001	0.001
Bleeding time prolonged	6	114.58 (51.23-256.29)	113.8 (667.42)	113.22 (50.62)	6.82 (1.63)	<0.001	<0.001
Aphasia	6	4.61 (2.07-10.3)	4.59 (16.86)	4.59 (2.06)	2.20 (0.50)	0.002	0.003
Iron deficiency anemia	5	13.17 (5.47-31.74)	13.10 (55.89)	13.1 (5.44)	3.71 (0.94)	<0.001	<0.001
Hepatotoxicity	5	14.12 (5.86-34.01)	14.04 (60.55)	14.03 (5.82)	3.81 (0.96)	<0.001	<0.001
Cholestasis	5	6.33 (2.63-15.26)	6.30 (22.32)	6.30 (2.62)	2.66 (0.56)	0.001	0.002
Transaminases increased	5	5.38 (2.23-12.95)	5.35 (17.71)	5.35 (2.22)	2.42 (0.45)	0.003	0.003
Dyslipidemia	5	23.26 (9.65-56.05)	23.13 (105.79)	23.11 (9.59)	4.53 (1.12)	<0.001	<0.001
Mental impairment	5	4.88 (2.03-11.76)	4.86 (15.33)	4.86 (2.02)	2.28 (0.38)	0.004	0.004
Peripheral coldness	5	8.64 (3.59-20.81)	8.59 (33.55)	8.59 (3.57)	3.10 (0.74)	<0.001	<0.001
Blood pressure inadequately controlled	5	18.88 (7.83-45.49)	18.77 (84.09)	18.76 (7.79)	4.23 (1.06)	<0.001	<0.001
Feeling drunk	4	12.61(4.72-33.69)	12.56 (42.54)	12.55 (4.70)	3.65 (0.63)	<0.001	<0.001
Low density lipoprotein increased	4	12.68 (4.75-33.87)	12.63 (42.81)	12.62 (4.72)	3.66 (0.63)	<0.001	<0.001
Drug level increased	4	5.69 (2.13-15.2)	5.67 (15.39)	5.67 (2.12)	2.50 (0.26)	0.006	0.006
Drug level below therapeutic	4	17.63 (6.6-47.11)	17.56 (62.43)	17.54 (6.57)	4.13 (0.73)	<0.001	<0.001
Endometrial hyperplasia	4	114.57 (42.79-306.74)	114.05 (445.92)	113.46 (42.38)	6.83 (0.97)	<0.001	<0.001
Amenorrhea	4	5.67 (2.12-15.14)	5.65 (15.31)	5.65 (2.11)	2.50 (0.25)	0.006	0.006
Febrile bone marrow aplasia	3	17.74 (5.71-55.13)	17.68 (47.18)	17.67 (5.68)	4.14 (0.33)	<0.001	<0.001
Central hypothyroidism	3	183.89 (58.91-573.98)	183.26 (539.27)	181.74 (58.22)	7.51 (0.52)	<0.001	<0.001
Malnutrition	3	6.57 (2.11-20.42)	6.55 (14.12)	6.55 (2.11)	2.71 (0.01)	0.011	0.011
Growth retardation	3	24.03 (7.73-74.71)	23.95 (65.92)	23.93 (7.7)	4.58 (0.38)	<0.001	<0.001
Intention tremor	3	128.58 (41.25-400.77)	128.14 (376.24)	127.40 (40.87)	6.99 (0.51)	<0.001	<0.001
Apraxia	3	48.44 (15.57-150.67)	48.27 (138.59)	48.17 (15.49)	5.59 (0.46)	<0.001	<0.001
Suspected product quality issue	3	47.09 (15.14-146.46)	46.93 (134.57)	46.83 (15.06)	5.55 (0.46)	<0.001	<0.001
Major depression	3	9.69 (3.12-30.10)	9.66 (23.28)	9.65 (3.11)	3.27 (0.16)	0.004	0.004
Breast pain	3	6.59 (2.12-20.46)	6.57 (14.16)	6.56 (2.11)	2.71 (0.01)	0.011	0.011
Hiccups	3	8.98 (2.89-27.89)	8.95 (21.18)	8.95 (2.88)	3.16 (0.14)	0.005	0.005
Abortion induced	3	7.42 (2.39-23.06)	7.40 (16.6)	7.40 (2.38)	2.89 (0.06)	0.008	0.008

*P* values were calculated using chi-square or Fisher’s exact tests based on 2×2 contingency tables. False discovery rate correction was performed using the Benjamini–Hochberg method, and q values represent adjusted p-values for multiple testing.

PT, Preferred Term; ROR, reporting odds ratio; CI, confidence interval; PRR, proportional reporting ratio; χ^2^, chi-squared; EBGM, empirical Bayesian geometric mean; EBGM05, the lower limit of the 95% confidence interval for EBGM; IC, information component; IC025, the lower limit of the 95% confidence interval for IC.

**Figure 3:**
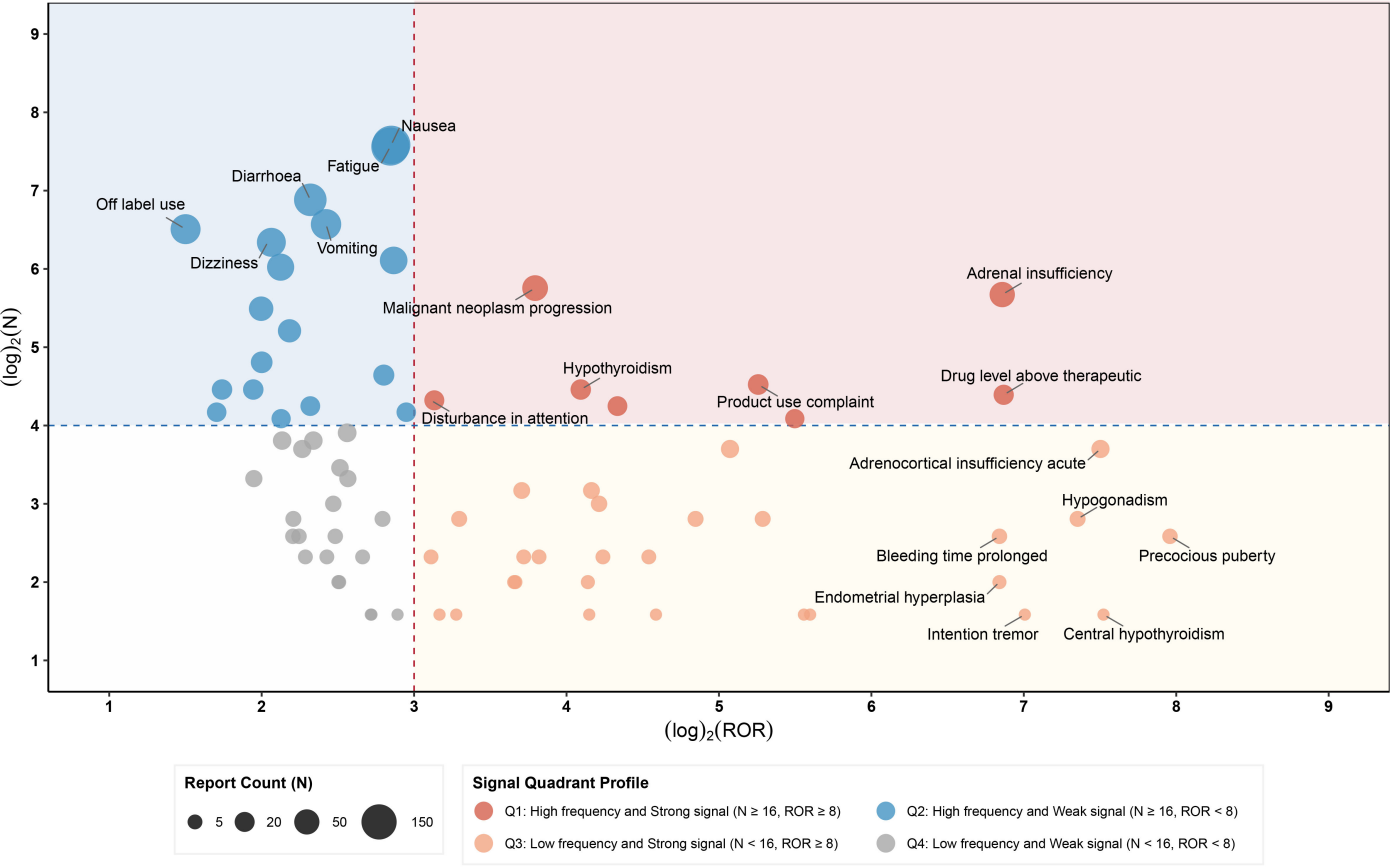
Quadrant bubble plot of mitotane-associated adverse event signals. Each bubble represents a preferred term (PT), with its size proportional to report counts (N). The x-axis and y-axis indicate log_2_-transformed ROR and report counts (N), respectively. Dashed lines represent the thresholds (log_2_ (ROR) = 3 and log_2_ (N) = 4) that divide the plot into four distinct color-coded quadrants (Q1-Q4). Text labels are selectively displayed for the top 6–7 prominent signals in each quadrant to ensure clarity.

Among these signals, the most frequently reported AE was nausea (n = 193), followed by fatigue (n = 189), diarrhea (n = 118), vomiting (n = 95), and off-label use (n = 91). Notably, several signals demonstrated marked disproportionality, most prominently precocious puberty (n = 6, ROR = 248.81, PRR = 247.10, EBGM = 244.35, IC = 7.93), central hypothyroidism (n = 3, ROR = 183.89, PRR = 183.26, EBGM = 181.74, IC = 7.51), acute adrenocortical insufficiency (n = 13, ROR = 181.38, PRR = 178.69, EBGM = 177.25, IC = 7.47), hypogonadism (n = 7, ROR = 163.47, PRR = 162.16, EBGM = 160.97, IC = 7.33), and intention tremor (n = 3, ROR = 128.58, PRR = 128.14, EBGM = 127.40, IC = 6.99). A bubble chart was utilized to visualize the signal strength. As shown in **Figure 3**, bubbles located in the upper-right quadrant represent AEs with both high reporting frequency and strong disproportionality signals. In contrast, those in the lower-left quadrant indicate AEs with relatively lower reporting frequency and weaker signal strength.

### Distribution of safety signals across SOC categories

**Figure 4:**
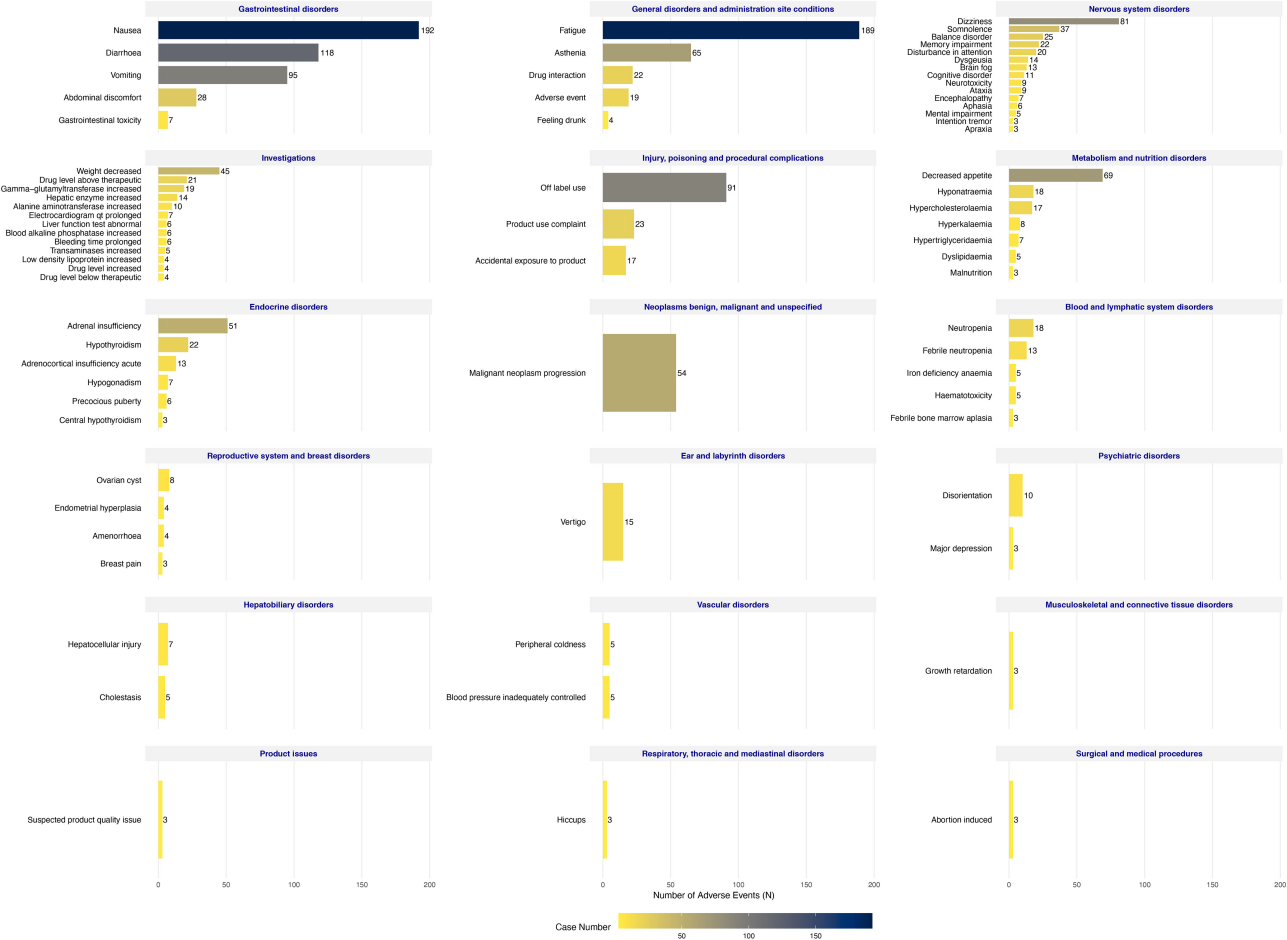
Distribution of mitotane-related safety signals across MedDRA System Organ Classes (SOCs). Each panel represents an individual SOC containing significant safety signals. The horizontal bars indicate the frequency of specific Preferred Terms (PTs) within the corresponding SOC. Only SOCs with identified positive signals are displayed.

To explore the organ-system level distribution of mitotane-related safety concerns, these 75 safety signals were mapped to 18 SOCs according to MedDRA classification. The SOC with the highest cumulative frequency was gastrointestinal disorders (n = 440), followed by general disorders and administration site conditions (n = 299), and nervous system disorders (n = 265). These findings indicate that gastrointestinal, systemic, and neurologic manifestations are the most prevalent AE categories associated with mitotane. Other SOCs with moderate frequencies included investigations (n = 151), injury, poisoning and procedural complications (n = 131), and metabolism and nutrition disorders (n = 127). Conversely, certain SOCs showed relatively low cumulative frequencies, such as musculoskeletal and connective tissue disorders (n = 3), respiratory, thoracic and mediastinal disorders (n = 3), and surgical and medical procedures (n = 3). The granular distribution of specific PTs within each corresponding SOC is illustrated in **Figure 4**, highlighting the diversity of toxicities associated with mitotane.

### Clinical prioritization of safety signals

Among the 75 safety signals identified for mitotane, only one (1.3%) was classified as a DME, whereas 16 (21.3%) were categorized as IMEs. Regarding the strength of supportive evidence, 46 signals (61.3%) were rated as “++” and 17 signals (22.7%) as “+”. On the basis of the clinical priority scoring system, 21 PTs (28.0%) were assigned moderate clinical priority, while the majority (54 PTs, 72.0%) were deemed to have low clinical priority. As shown in **Table 4**, adrenal insufficiency emerged as the highest-priority signal, achieving a score of 7 points, underscoring its critical clinical relevance. Several other AEs demonstrated relatively high prioritization (6 points each), including nausea, fatigue, decreased appetite, malignant neoplasm progression, hypothyroidism, febrile neutropenia, and acute adrenocortical insufficiency.

**Table 4:** Clinical priority assessing results of safety signals.

PT	N	Lower limit of the 95% CI for ROR	Mortality proportion	DMEs/IMEs	Relevant evidence evaluation	Priority level (score)
Nausea	192	6.15	2.08%		++	moderate (6)
Fatigue	189	6.12	4.23%		++	moderate (6)
Diarrhea	118	4.11	1.69%		++	moderate (5)
Vomiting	95	4.33	4.21%		++	moderate (5)
Off-label use	91	2.28	6.59%		–	weak (3)
Dizziness	81	3.32	1.23%		++	moderate (5)
Decreased appetite	69	5.7	4.35%		++	moderate (6)
Asthenia	65	3.39	4.62%		++	moderate (5)
Malignant neoplasm progression	54	10.53	25.93%	IME	–	moderate (6)
Adrenal insufficiency	51	87.39	3.92%	IME	++	moderate (7)
Weight decreased	45	2.96	0.00%		+	weak (3)
Somnolence	37	3.26	0.00%		++	weak (4)
Abdominal discomfort	28	2.74	0.00%		++	weak (4)
Balance disorder	25	4.68	0.00%		++	weak (4)
Product use complaint	23	25.27	0.00%		–	weak (3)
Hypothyroidism	22	11.18	9.09%	IME	++	moderate (6)
Drug interaction	22	2.19	4.55%		–	weak (2)
Memory impairment	22	2.52	0.00%		++	weak (4)
Drug level above therapeutic	21	75.68	4.76%		–	weak (3)
Disturbance in attention	20	5.63	0.00%		++	moderate (5)
Adverse event	19	3.17	0.00%		–	weak (2)
Gamma-glutamyl transferase increased	19	12.8	0.00%		++	moderate (5)
Neutropenia	18	2.04	11.11%	IME	++	moderate (5)
Hyponatremia	18	4.84	5.56%		++	weak (4)
Accidental exposure to product	17	2.7	0.00%		–	weak (2)
Hypercholesterolemia	17	27.94	5.88%		++	moderate (5)
Vertigo	15	3.54	0.00%		++	weak (4)
Hepatic enzyme increased	14	2.99	0.00%		++	weak (4)
Dysgeusia	14	2.59	0.00%		++	weak (4)
Febrile neutropenia	13	2.78	15.38%	DME	++	moderate (6)
Adrenocortical insufficiency acute	13	104.66	0.00%	IME	++	moderate (6)
Brain fog	13	19.45	0.00%		++	moderate (5)
Cognitive disorder	11	3.15	0.00%		++	weak (4)
Alanine aminotransferase increased	10	2.07	0.00%		++	weak (4)
Disorientation	10	3.17	0.00%		+	weak (3)
Neurotoxicity	9	6.76	0.00%	IME	++	moderate (5)
Ataxia	9	9.29	0.00%		++	weak (4)
Hyperkalemia	8	2.76	12.50%	IME	+	weak (3)
Ovarian cyst	8	9.24	0.00%		++	weak (4)
Hypogonadism	7	77.48	0.00%		++	weak (4)
Gastrointestinal toxicity	7	18.54	0.00%		++	weak (4)
Hepatocellular injury	7	4.67	28.57%	IME	++	moderate (5)
Electrocardiogram QT prolonged	7	2.2	0.00%	IME	+	weak (3)
Hypertriglyceridemia	7	13.67	14.29%		++	weak (4)
Encephalopathy	7	3.29	14.29%	IME	+	weak (3)
Precocious puberty	6	110.97	0.00%		+	weak (3)
Liver function test abnormal	6	2.12	0.00%		++	weak (3)
Blood alkaline phosphatase increased	6	2.5	0.00%		++	weak (3)
Bleeding time prolonged	6	51.23	0.00%		++	weak (4)
Aphasia	6	2.07	0.00%		++	weak (3)
Iron deficiency anemia	5	5.47	0.00%		+	weak (3)
Hepatotoxicity	5	5.86	40.00%		++	moderate (5)
Cholestasis	5	2.63	0.00%	IME	++	weak (4)
Transaminases increased	5	2.23	20.00%		++	weak (3)
Dyslipidemia	5	9.65	0.00%		++	weak (4)
Mental impairment	5	2.03	0.00%	IME	++	weak (4)
Peripheral coldness	5	3.59	0.00%		+	weak (2)
Blood pressure inadequately controlled	5	7.83	0.00%		+	weak (3)
Feeling drunk	4	4.72	0.00%		+	weak (2)
Low density lipoprotein increased	4	4.75	0.00%		++	weak (3)
Drug level increased	4	2.13	0.00%		–	weak (1)
Drug level below therapeutic	4	6.6	25.00%		–	weak (3)
Endometrial hyperplasia	4	42.79	0.00%		+	weak (3)
Amenorrhea	4	2.12	0.00%		++	weak (3)
Febrile bone marrow aplasia	3	5.71	0.00%	IME	–	weak (3)
Central hypothyroidism	3	58.91	0.00%	IME	++	moderate (5)
Malnutrition	3	2.11	33.33%		+	weak (3)
Growth retardation	3	7.73	0.00%	IME	+	weak (4)
Intention tremor	3	41.25	0.00%		+	weak (3)
Apraxia	3	15.57	0.00%		+	weak (3)
Suspected product quality issue	3	15.14	0.00%		–	weak (2)
Major depression	3	3.12	0.00%	IME	++	weak (4)
Breast pain	3	2.12	0.00%		+	weak (2)
Hiccups	3	2.89	0.00%		+	weak (2)
Abortion induced	3	2.39	33.33%		–	weak (2)

PT, Preferred Term; ROR, reporting odds ratio; CI, confidence interval; IME, important medical events; DME, designated medical event.

### Time to onset analysis

**Figure 5:**
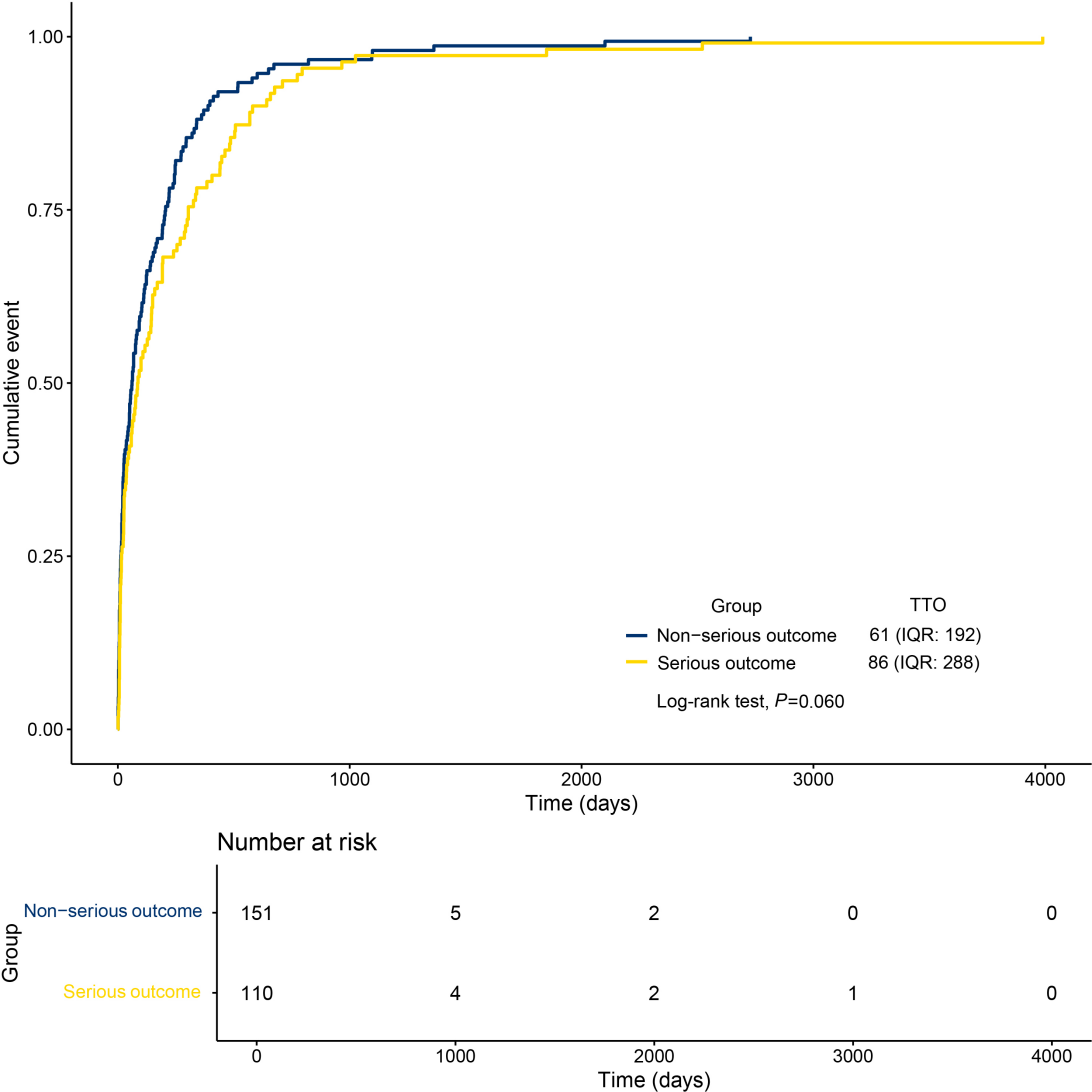
Distribution of time to onset (TTO) of mitotane-related adverse events stratified by seriousness. Kaplan-Meier curves were used to visualize the empirical distribution of TTO among reports with serious and non-serious outcomes. Group comparisons were performed using the log-rank test.

**Figure 6:**
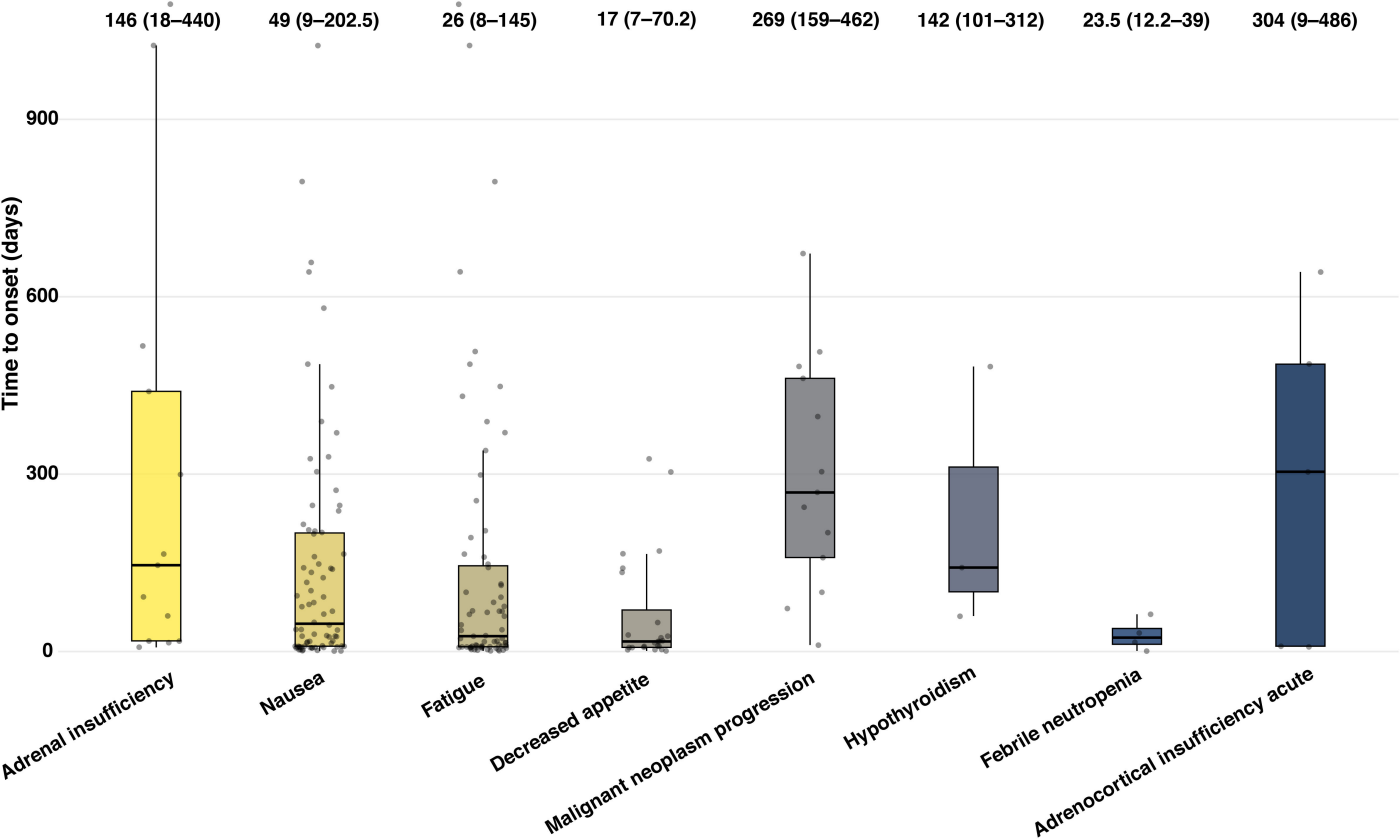
Time to onset (TTO) of adverse events with moderate clinical priority (score ≥ 6). Box-and-scatter plots depict the distribution of TTO for selected adverse events classified as moderate clinical priority, highlighting the heterogeneity in onset timing across different event types.

Of the 870 cases, TTO was calculable in 261 (30%). The median TTO was 68 (15-244) days. As shown in **Figure 5**, the median onset time for patients with serious outcomes was 86 (16.5-304) days, compared with 61 (13.5-205) days for those without serious outcomes. Although TTO appeared slightly longer in the serious outcome group, the difference was not statistically significant (log-rank test, *P* = 0.060). However, endocrine AEs exhibited a significantly longer TTO distribution compared with non-endocrine AEs (median 144 [28.5-472] vs 66 [9.5-205] days; log-rank test, *P* = 0.043). The TTO of AEs with moderate clinical priority (score ≥ 6) is illustrated in **Figure 6**. The median TTO values were as follows: adrenal insufficiency, 146 (18-440) days; nausea, 49 (9-202.5) days; fatigue, 26 (8-145) days; decreased appetite, 17 (7-70.2) days; malignant neoplasm progression, 269 (159-462) days; hypothyroidism, 142 (101-312) days; febrile neutropenia, 23.5 (12.2-39) days; and acute adrenocortical insufficiency, 304 (9-486) days.

### Machine learning-based characterization of serious AE patterns

While descriptive comparisons revealed several differences between reports with serious and non-serious outcomes, univariable analyses were insufficient to capture the complex associations between characteristics and outcomes. Therefore, a regularized logistic regression model with elastic net penalization and an XGBoost model were employed to characterize multivariable patterns associated with serious AEs using both linear and nonlinear modeling approaches, respectively.

**Figure 7:**
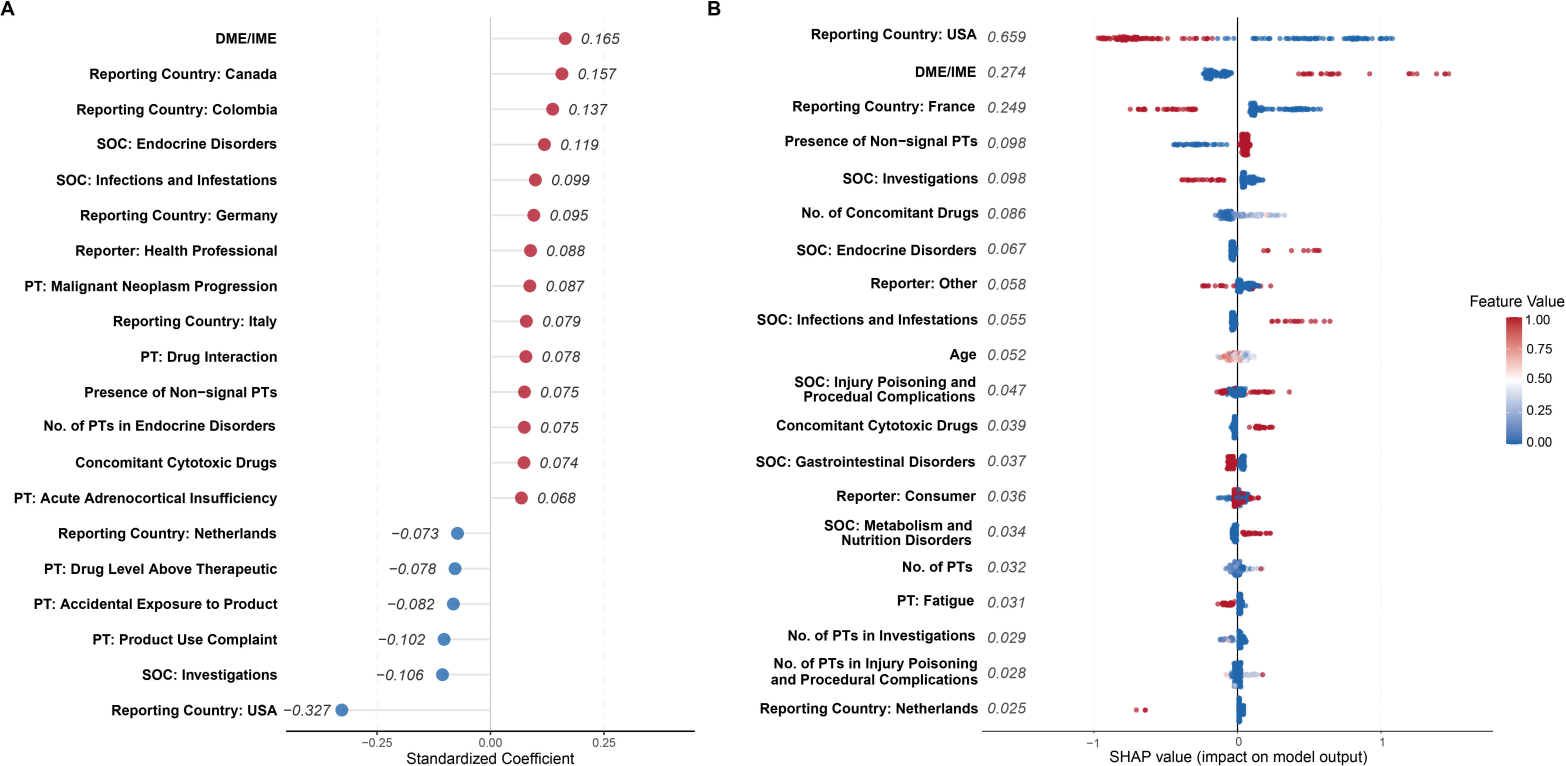
Multidimensional characterization of clinical and demographic features associated with serious outcomes. **(A)** Lollipop plot of standardized coefficients for the top 20 features derived from the elastic net logistic regression model. The position and color of each dot represent the strength and direction of the multivariable association. **(B)** SHAP (SHapley Additive exPlanations) summary plot for the top 20 features derived from the Extreme Gradient Boosting (XGBoost) model. Each point represents an individual report, with its position on the x-axis quantifying the feature’s contribution to the serious outcome pattern.

The elastic net logistic regression model identified multiple factors associated with report seriousness. As a descriptive measure of model discrimination, the mean 10-fold cross-validation AUROC was 0.830 (95% CI: 0.803-0.857). Among features with non-zero standardized coefficients, reporting country indicators and DME/IME status were prominently represented. SOC-level AE categories, including endocrine disorders, investigations, and infections and infestations, contributed significantly to the multivariable pattern. At the PT level, malignant neoplasm progression, accidental exposure to product, and product use complaint showed notable effects. In addition, health professional as reporter type and concomitant cytotoxic drug use were also important contributors to the model output (**Figure 7A**).

To explore potential nonlinear relationships and interactions, XGBoost with SHAP analysis was applied. The mean 10-fold cross-validation AUROC was 0.869 (95% CI: 0.858-0.879). As shown in **Figure 7B**, SHAP-based feature attribution highlighted reporting country indicators and DME/IME status as major contributors to the seriousness pattern. SOC-level AE categories, including investigations, endocrine disorders, infections and infestations, together with indicators reflecting medication complexity (such as the number of concomitant drugs and concomitant cytotoxic drug use), contributed to the multivariable pattern. Additionally, age, reporter type, and the number of reported PTs also showed notable contributions to the overall seriousness pattern.

Despite differences in model structure and feature attribution methods, the outputs from the two models exhibited a strong positive correlation (Pearson’s *r* = 0.881), indicating a high degree of concordance in the multivariable patterns identified.

### Sensitivity analyses

To assess the potential influence of concomitant medications on signal detection, a sensitivity analysis was performed using a restricted subset of 298 reports in which mitotane was recorded as the sole primary suspect drug. Repeating the disproportionality analysis identified 25 PT-level safety signals in the monotherapy subset. After Benjamini-Hochberg false discovery rate correction, 22 signals remained statistically significant (q < 0.05). Among these, 21 signals (95.5%) overlapped with those detected in the primary analysis, indicating a high degree of concordance between the two analyses. The only signal identified exclusively in the monotherapy subset was oral candidiasis (n = 3). Detailed results of the sensitivity analysis are presented in **Supplementary Table 4**.

To assess the potential influence of low-frequency AEs on the machine learning analyses, PT-level features reported in fewer than 10 cases were excluded and both modeling frameworks were rebuilt using the reduced feature set. Comparison of feature importance profiles demonstrated substantial stability after exclusion of low-frequency PT features. Among the top 20 variables identified by each model, 19 were retained from the primary analysis, corresponding to a feature overlap of 95% for both the elastic net and XGBoost frameworks. Although minor changes in variable ranking were observed, the overall multivariable association patterns remained largely unchanged. Consistent with this finding, model performance showed minimal variation, with mean 10-fold cross-validation AUROCs of 0.831 (95% CI: 0.804-0.858) for the elastic net model and 0.867 (95% CI: 0.857-0.878) for the XGBoost model. Detailed results are presented in **Supplementary Figure 1**.

## Discussion

This large-scale real-world pharmacovigilance study comprehensively characterized the safety profile of mitotane by integrating disproportionality analysis, clinical priority scoring, TTO analysis, and multivariable machine learning. Our findings confirmed established toxicities, particularly involving the gastrointestinal, neurological, endocrine, and metabolic systems, while also identifying less frequently reported but potentially clinically relevant AE patterns. The integration of conventional pharmacovigilance approaches with interpretable machine learning provided complementary insights into factors associated with serious outcomes.

Among 870 AE reports, a higher proportion occurred in patients aged 18–64 years, consistent with the peak incidence of ACC (17). AEs were more frequently reported in females (65.0%), likely reflecting the higher prevalence of ACC in women, as previously described (2). Nearly half of the reports were serious, with hospitalization and death accounting for a substantial proportion, underscoring the significant clinical burden of mitotane therapy. Stratified analyses revealed differences between serious and non-serious reports with respect to sex, age, reporting country, and reporter type, suggesting that these report-level characteristics are associated with the observed heterogeneity in seriousness pattern.

Disproportionality analyses consistently identified strong safety signals across multiple statistical methods. Gastrointestinal disorders emerged as the most frequently reported AEs, particularly nausea, diarrhea, and vomiting, likely due to mitotane’s lipophilicity and local gastrointestinal exposure. These AEs are well-recognized contributors to treatment intolerance and reduced adherence (32). To mitigate these AEs, clinical strategies such as fractional dosing and co-administration with lipid-rich foods are commonly used to improve tolerability.

Nervous system disorders were the second most prominent category and were typically associated with elevated plasma concentrations, particularly above 14–20 mg/L (33–35). Common manifestations such as dizziness, somnolence, and cognitive impairment are well documented and often dose-limiting (8). While substantially impacting quality of life during treatment, the neurotoxicity is generally reversible upon dose reduction or discontinuation (36).

Endocrine disorders, especially adrenal insufficiency and hypothyroidism, ranked highest in clinical priority. Adrenal insufficiency achieved the highest composite priority score, reflecting its frequency, seriousness, and strong causal plausibility. Unlike adrenal insufficiency from other etiologies, mitotane-associated adrenal insufficiency occurs frequently and may require prophylactic management with higher doses of glucocorticoids (37–39). Inadequate replacement may exacerbate toxicity and compromise treatment tolerance (40). Hypothyroidism and hypogonadism, which are also frequently observed, further illustrate the broad endocrine-disrupting properties of mitotane (41, 42). Precocious puberty was observed in six pediatric patients aged 1–5 years. Although this reaction is not currently listed in the official mitotane product labeling, several case reports have documented its occurrence during mitotane therapy (43, 44). The underlying mechanism may involve the estrogenic activity of mitotane, mediated by estrogen receptor α activation and increased hepatic synthesis of sex hormone-binding globulin (45, 46). Furthermore, mitotane may act as an estrogen receptor α agonist, promoting estrogen-like effects and accelerating bone age maturation (44).

Metabolic and nutritional disturbances, particularly dyslipidemia manifesting as hypercholesterolemia and hypertriglyceridemia, were also identified. Mitotane therapy is associated with clinically significant elevations in low-density lipoprotein cholesterol (LDL-C), high-density lipoprotein cholesterol (HDL-C), triglycerides, and non-HDL-C (47–49). These effects are attributed to the inhibition of cytochrome P450 enzymes, which impairs oxysterol formation and upregulates HMG-CoA reductase activity, thereby enhancing cholesterol synthesis (50). Current guidelines recommend monitoring lipid profiles every 3–4 months, with statin therapy (preferably rosuvastatin or pravastatin, as they are not metabolized by CYP3A4) initiated if dyslipidemia persists (15). Hyponatremia, a reaction not currently listed in the official mitotane product labeling, was identified as a safety signal and reported in 13 patients. Among these cases, four occurred concurrently with adrenal insufficiency, one with hypertriglyceridemia, and two with hyperkalemia. Hormonal deficiency, including primary or secondary adrenal insufficiency and severe hypothyroidism, may contribute to the development of hypotonic hyponatremia (48). Additionally, severe hypertriglyceridemia is associated with clinically significant pseudohyponatremia, potentially resulting in underestimation of actual serum sodium levels (49).

Reproductive and gynecological AEs, including ovarian cysts and menstrual disturbances, were frequently observed in women. Previous studies have indicated that mitotane impairs ovarian steroidogenesis and disrupts pituitary-ovarian feedback, which could contribute to the development of functional ovarian cysts and menstrual disturbances in premenopausal women, typically within the first year of treatment and independent of plasma concentration (51, 52). These effects are generally benign and reversible upon treatment discontinuation. Accordingly, regular gynecological monitoring is recommended, and hormonal contraception may help reduce cyst formation (51, 53–56). Endometrial hyperplasia and abnormal uterine bleeding likely reflect mitotane-associated endocrine imbalance, particularly relative hyperestrogenism, and may be aggravated by drug-related platelet dysfunction (57, 58). In our analysis, endometrial hyperplasia, prolonged bleeding time, and anemia emerged as notable safety signals. Severe or chronic bleeding may contribute to the development of secondary anemia, which is consistent with findings from the ADIUVO trial (20). Sustained progestogen therapy has been reported to alleviate these symptoms, and the use of a levonorgestrel-releasing intrauterine device was found to be effective and well tolerated in patients with menorrhagia (59).

Hematologic toxicity is predominantly mild, with leukopenia and anemia most frequently reported (60). In our analysis, most cases of neutropenia and febrile neutropenia occurred in patients receiving concomitant cytotoxic chemotherapy (e.g., the EDP regimen: etoposide, doxorubicin, and cisplatin), which is the standard of care for advanced ACC (61). These observations suggest that severe hematologic events may be influenced, at least in part, by concomitant medications rather than mitotane alone. This interpretation was further supported by the monotherapy-based sensitivity analysis, in which febrile neutropenia was no longer detected as a significant disproportionality signal. Consistent with these findings, the XGBoost model identified concomitant cytotoxic drug use as an important variable associated with serious outcomes. Bone marrow aplasia was rare and was observed exclusively in patients treated with combined cytotoxic regimens, further supporting a limited contribution of mitotane to such severe hematologic toxicities. Nevertheless, given the prolonged half-life and cumulative exposure of mitotane, regular hematologic monitoring and timely dose adjustment remain essential components of safe treatment (15, 20).

Hepatotoxicity represents another relevant AE associated with mitotane therapy. Elevations in hepatic enzymes are common, with γ-glutamyl transpeptidase (GGT) levels increased in nearly all patients, while alanine transaminase (ALT) and aspartate transaminase (AST) elevations occur less frequently (62–64). These abnormalities are generally reversible following dose reduction or treatment discontinuation (38, 65). Consistent with these clinical observations, hepatotoxicity and abnormal liver function tests emerged as safety signals in our analysis, underscoring the importance of regular liver function monitoring during mitotane treatment.

By incorporating signal strength, seriousness, mortality proportion, and existing evidence, the clinical priority assessment facilitated a more clinically meaningful interpretation of pharmacovigilance signals. While gastrointestinal and neurological AEs were highly prevalent, endocrine disorders were prioritized due to their potential for rapid clinical deterioration if unrecognized, which can lead to life-threatening adrenal crises. Conversely, less frequent events, such as hepatocellular injury and hematologic abnormalities, were assigned moderate priority, emphasizing the importance of considering clinical impact rather than frequency alone.

TTO analysis provided additional descriptive information on the temporal distribution of AEs. Non-endocrine events, such as nausea, decreased appetite, and fatigue, tended to occur earlier after treatment initiation, suggesting short-term intolerance rather than cumulative toxicity. These findings are consistent with pharmacovigilance data demonstrating early signals for non-endocrine AEs (42). In contrast, endocrine disorders showed relatively longer TTO distributions. Their median onset times substantially exceeded those of non-endocrine AEs, consistent with previous evidence of delayed mitotane-associated endocrine dysfunction (66). Such differences likely reflect the drug’s slow attainment of steady state, tissue accumulation, and progressive interference with endocrine homeostasis (67).

To further explore factors associated with serious outcomes, we applied Elastic Net regression and XGBoost modeling to the FAERS data. Both approaches yielded broadly consistent findings and identified similar multivariable patterns associated with serious outcomes. In both frameworks, report-related indicators emerged as major contributors. Features including reporting country indicators and DME/IME status reflected intrinsic structural attributes of pharmacovigilance reporting rather than isolated clinical events. It should be noted that the high feature importance of reporting country reflects systemic reporting biases and regulatory heterogeneity rather than genuine pharmacological risk or intrinsic drug toxicity. Therefore, these non-clinical attributes must not be interpreted as clinical risk factors. In addition, AE information aggregated at the SOC level, such as endocrine disorders, investigations, and infections and infestations, was consistently associated with seriousness, suggesting that broader system-level toxicity profiles provide critical contextual information. At the PT level, malignant neoplasm progression, product use complaint, and acute adrenocortical insufficiency showed notable associations in the Elastic Net model, while fatigue also contributed within the XGBoost framework. Indicators of report and medical complexity, including the overall concomitant medication burden, the specific use of cytotoxic agents, and the total diversity of reported events (the number of reported PTs), further shaped the seriousness profile. Reporter occupation type also contributed marginally to the model outputs. The two analytical approaches yielded consistent multivariable association patterns, supporting the robustness of the findings across different modeling frameworks.

A major strength of this study lies in the use of the FAERS database to evaluate mitotane safety in a real-world setting, particularly given the rarity of ACC. By combining signal detection, clinical priority assessment, TTO evaluation, and machine learning, we were able to examine different dimensions of reported AEs beyond frequency alone. The concordance between traditional and model-based approaches enhances the robustness of our findings. However, several limitations should be acknowledged. First, FAERS is a spontaneous reporting system subject to underreporting, reporting bias, missing data, and the absence of exposure denominators. Consequently, incidence rates cannot be estimated, and the identified signals should be interpreted as hypothesis-generating associations rather than evidence of causality. Second, residual confounding by disease severity and concomitant medications cannot be fully excluded. In addition, although the machine learning analyses provide complementary insights into factors associated with report seriousness, they were designed as exploratory analytical approaches rather than clinically deployable prediction models. External validation was not performed, and the identified variables should be interpreted as factors associated with reporting patterns within FAERS rather than causal determinants. Third, the clinical priority scoring system is a semi-quantitative framework with equal weighting across dimensions. While this approach ensures methodological transparency and consistency with prior pharmacovigilance studies, it remains an arbitrary prioritization tool rather than a formally validated or parametric classification model. Accordingly, the resulting prioritization should be interpreted with caution, and alternative weighting strategies warrant future investigation. Despite these limitations, FAERS remains a valuable real-world pharmacovigilance resource, enabling the detection of rare or delayed AEs in orphan diseases and complementing evidence from clinical trials (23, 68).

## Conclusion

This study provides a comprehensive real-world safety assessment of mitotane by integrating signal detection, clinical priority evaluation, TTO analyses, and interpretable machine learning. Through both elastic net and XGBoost modeling, we identified consistent multivariable patterns associated with serious outcomes, reflecting reporting country, medical event designation, organ system involvement, and medication complexity. These findings support the internal consistency of the combined analyses and highlight their value in refining the interpretation of pharmacovigilance signals.

## Data Availability

The raw data supporting the conclusions of this article will be made available by the authors, without undue reservation.
