# Supplementary material for: Real-world safety profile of mitotane in adrenocortical carcinoma: a retrospective cross-sectional study integrating pharmacovigilance and interpretable machine learning
Source: Front Endocrinol (Lausanne). 2026 Jul 22;17:1862262. doi: 10.3389/fendo.2026.1862262 (PMC13437372; doi:10.3389/fendo.2026.1862262)
Supplement: Supplementary file 1 [file Table1.docx]

**Supplemental Table 1** The formulas of disproportionate measurement and the criteria for signal detection.

| **Method** | **Calculation formula** | **Criteria** |
| --- | --- | --- |
| ROR | $ROR=\frac{a / c}{b / d}$ | a ≥ 3  95%CI (lower limit) > 1 |
|  | $SE(lnROR)=\sqrt{\frac{1}{a}+\frac{1}{b}+\frac{1}{c}+\frac{1}{d}}$ |  |
|  | $95\%CI= e^{\ln\left( ROR \right)\pm1.96se}$ |  |
| PRR | $PRR=\frac{a / (a+b)}{c / (c+d)}$ | a ≥ 3  PRR ≥ 2  **χ^2^** ≥ 4 |
|  | $SE(lnPRR)=\sqrt{\frac{1}{a}-\frac{1}{a+b}+\frac{1}{c}-\frac{1}{c+d}}$ |  |
|  | $95\%CI= e^{\ln\left( PRR \right)\pm1.96se}$ |  |
|  | $\chi2 =\frac{{(ad-bc)}^{2}(a+b+c+d)}{( a+b)(a+c)(c+d)(b+d)}$ |  |
| BCPNN | IC=${log}_{2}\frac{p(x,y)}{p(x)p(y)}={log}_{2}\frac{a(a+b+c+d)}{(a+b)(a+c)}$ | IC025 > 0 |
|  | E(IC)=${log}_{2}\frac{(a+\gamma11)(a+b+c+d+\alpha)(a+b+c+d+\beta)}{（a+b+c+d+\gamma）(a+b+\alpha1)(a+c+\beta1)}$ |  |
|  | $V\left( IC \right)=\frac{1}{{(ln2)}^{2}}\{\left[ \frac{\left( a+b+c+d \right)-a+\gamma-\gamma11}{\left( a+\gamma11 \right)\left( 1+a+b+c+d+\gamma\right)} \right]+\left[ \frac{\left( a+b+c+d \right)-\left( a+b \right)+\alpha-\alpha1}{\left( a+b+\alpha1 \right)\left( 1+a+b+c+d+\alpha\right)} \right]+\left[ \frac{\left( a+b+c+d \right)-\left( a+c \right)+\beta-\beta1}{\left( a+c+\beta1 \right)\left( 1+a+b+c+d+\beta\right)} \right]\}$ |  |
|  | $\gamma=\gamma11\frac{(a+b+c+d+\alpha)(a+b+c+d+\beta)}{(a+b+\alpha1)(a+c+\beta1)}$ |  |
|  | *IC-2SD=E(IC)-2*$\sqrt{V(IC)}$  $\alpha1=\beta1=1, \alpha=\beta=2, \gamma11=1$ |  |
| EBGM | $EBGM=\frac{a(a+b+c+d)}{\left( a+c \right)(a+b)}$ | EBGM05 > 2 |
|  | $SE(lnEBGM)=\sqrt{\frac{1}{a}+\frac{1}{b}+\frac{1}{c}+\frac{1}{d}}$ |  |
|  | $95\%CI= e^{\ln\left( EBGM \right)\pm1.96se}$ |  |

Note: ^a^ number of reports with target AE and mitotane; ^b^ number of reports with mitotane without target AE; ^c^ number of reports with target AE without mitotane; ^d^ number of reports without target AE and mitotane.

Abbreviations: ROR, reporting odds ratio; CI, confidence interval; PRR, proportional reporting ratio; χ^2^, chi-squared; BCPNN, Bayesian confidence propagation neural network; IC, information component; IC025, lower limit of the 95% confidence interval for IC; E (IC), expected value of the IC; V (IC), the variance of IC; EBGM, empirical Bayesian geometric mean; EBGM05, the lower limit of the 95% confidence interval for EBGM.

**Supplemental Table 2** A semi-quantitative scoring system assessing clinical priority of safety signals.

| **Clinical priority features** | **2 points** | **1 point** | **0 point** |
| --- | --- | --- | --- |
| Number of target events | >50 | 10-50 | <10 |
| the lower limit of the 95% CI for ROR | >5 | 2-5 | 1-2 |
| Mortality proportion | >50% | 25%-50% | <25% |
| IMEs or DMEs | DME | IME | None |
| Relevant evidence evaluation | ++ | + | **-** |

Note: ^++^ adverse events are mainly from the FDA Prescribing Information, the summary of product characteristics of mitotane posted by the Medicines and Healthcare Products Regulatory Agency, Phase 2/3 random control trials, or systematic reviews, with biological plausibility; ^+^ AEs are mainly from other clinical trials, observational studies, or case reports/series with potential biological plausibility; ^-^ AEs only emerging from disproportionality analyses.

Abbreviations: CI, confidence interval; ROR, reporting odds ratio; IME, important medical events; DME, designated medical event.

**Supplemental Table 3** Distribution of adverse events in mitotane-related reports stratified by outcome seriousness.

| **PT**  **(Top 30 by serious reports)** | **Serious**  **outcome group  (N=398)** | **Non-serious outcome group  (N=472)** | ***P*** |
| --- | --- | --- | --- |
| Fatigue | 74 (18.6%) | 115 (24.4%) | 0.047 |
| Nausea | 74 (18.6%) | 118 (25.0%) | 0.027 |
| Off label use | 48 (12.1%) | 43 (9.1%) | 0.182 |
| Malignant neoplasm progression | 46 (11.6%) | 8 (1.7%) | <0.001 |
| Vomiting | 46 (11.6%) | 49 (10.4%) | 0.587 |
| Adrenal insufficiency | 39 (9.8%) | 12 (2.5%) | <0.001 |
| Diarrhea | 39 (9.8%) | 79 (16.7%) | 0.003 |
| Asthenia | 38 (9.5%) | 27 (5.7%) | 0.038 |
| Dizziness | 33 (8.3%) | 48 (10.2%) | 0.352 |
| Decreased appetite | 30 (7.5%) | 39 (8.3%) | 0.708 |
| Death | 23 (5.8%) | 7 (1.5%) | <0.001 |
| Weight decreased | 22 (5.5%) | 23 (4.9%) | 0.759 |
| Drug ineffective | 19 (4.8%) | 11 (2.3%) | 0.061 |
| Drug interaction | 19 (4.8%) | 3 (0.6%) | <0.001 |
| Headache | 19 (4.8%) | 22 (4.7%) | 1.000 |
| Hypothyroidism | 19 (4.8%) | 3 (0.6%) | <0.001 |
| Somnolence | 17 (4.3%) | 20 (4.2%) | 1.000 |
| Confusional state | 14 (3.5%) | 7 (1.5%) | 0.074 |
| Neutropenia | 14 (3.5%) | 4 (0.8%) | 0.007 |
| Pyrexia | 14 (3.5%) | 4 (0.8%) | 0.007 |
| Adrenocortical insufficiency acute | 13 (3.3%) | 0 (0.0%) | <0.001 |
| Disturbance in attention | 13 (3.3%) | 7 (1.5%) | 0.111 |
| Hyponatraemia | 13 (3.3%) | 5 (1.1%) | 0.030 |
| Memory impairment | 13 (3.3%) | 9 (1.9%) | 0.278 |
| Hypercholesterolemia | 12 (3.0%) | 5 (1.1%) | 0.048 |
| Balance disorder | 11 (2.8%) | 14 (3.0%) | 1.000 |
| Febrile neutropenia | 11 (2.8%) | 2 (0.4%) | 0.009 |
| Anemia | 10 (2.5%) | 6 (1.3%) | 0.209 |
| Gastrointestinal disorder | 10 (2.5%) | 6 (1.3%) | 0.209 |
| Vertigo | 10 (2.5%) | 5 (1.1%) | 0.120 |

Abbreviations: PT, Preferred Term.

**Supplemental Table 4** Sensitivity analysis of disproportionality signals in the mitotane monotherapy subset.

| **PT** | **N** | **ROR (95%Cl)** | **PRR (χ^2^)** | **EBGM (EBGM05)** | **IC (IC025)** | ***P* value** | **q value** |
| --- | --- | --- | --- | --- | --- | --- | --- |
| Nausea | 45 | 4.53 (3.30–6.22) | 4.00 (101.98) | 4.04 (3.02) | 2.01 (1.72) | <0.001 | <0.001 |
| Fatigue | 38 | 3.78 (2.69–5.32) | 3.43 (65.37) | 3.47 (2.53) | 1.80 (1.48) | <0.001 | <0.001 |
| Vomiting | 23 | 3.66 (2.39–5.60) | 3.45 (38.52) | 3.53 (2.34) | 1.82 (1.41) | <0.001 | <0.001 |
| Accidental exposure to product | 17 | 13.25 (8.12–21.62) | 12.55 (170.12) | 12.92 (8.03) | 3.69 (3.22) | <0.001 | <0.001 |
| Malignant neoplasm progression | 9 | 6.52 (3.36–12.67) | 6.36 (35.61) | 6.71 (3.49) | 2.75 (2.09) | <0.001 | <0.001 |
| Product use complaint | 8 | 38.82 (19.23–78.38) | 37.80 (251.05) | 40.14 (20.08) | 5.33 (4.63) | <0.001 | <0.001 |
| Ovarian cyst | 8 | 55.14 (27.31–111.34) | 53.69 (362.51) | 56.99 (28.50) | 5.83 (5.14) | <0.001 | <0.001 |
| Hypothyroidism | 8 | 18.14 (8.98–36.62) | 17.68 (109.89) | 18.78 (9.39) | 4.23 (3.54) | <0.001 | <0.001 |
| Adrenal insufficiency | 7 | 44.63 (21.08–94.48) | 43.61 (250.32) | 46.69 (22.26) | 5.55 (4.80) | <0.001 | <0.001 |
| Drug level above therapeutic | 7 | 113.21 (53.46–239.73) | 110.57 (653.44) | 118.26 (56.38) | 6.89 (6.15) | <0.001 | <0.001 |
| Adverse event | 6 | 4.59 (2.05–10.30) | 4.52 (13.16) | 4.89 (2.20) | 2.29 (1.49) | 0.002 | 0.039 |
| Neurotoxicity | 6 | 25.65 (11.43–57.56) | 25.15 (116.08) | 27.23 (12.24) | 4.77 (3.97) | <0.001 | <0.001 |
| Gastrointestinal toxicity | 6 | 98.88 (44.03–222.04) | 96.91 (477.01) | 104.83 (47.09) | 6.71 (5.91) | <0.001 | <0.001 |
| Vertigo | 5 | 5.74 (2.37–13.90) | 5.66 (14.86) | 6.23 (2.59) | 2.64 (1.76) | 0.002 | 0.035 |
| Bleeding time prolonged | 5 | 281.33 (116.01–682.26) | 276.63 (1106.64) | 302.98 (126.11) | 8.24 (7.37) | <0.001 | <0.001 |
| Disturbance in attention | 4 | 5.07 (1.89–13.60) | 5.02 (9.18) | 5.64 (2.12) | 2.50 (1.52) | 0.009 | 0.100 |
| Hypercholesterolemia | 4 | 30.80 (11.48–82.63) | 30.40 (86.23) | 34.18 (12.83) | 5.10 (4.12) | <0.001 | <0.001 |
| Amenorrhea | 4 | 16.70 (6.23–44.80) | 16.49 (43.76) | 18.55 (6.96) | 4.21 (3.23) | <0.001 | 0.003 |
| Endometrial hyperplasia | 3 | 251.93 (80.61–787.35) | 249.40 (512.61) | 289.84 (93.48) | 8.18 (7.05) | <0.001 | <0.001 |
| Suspected product quality issue | 3 | 138.39 (44.32–432.09) | 137.01 (279.88) | 159.50 (51.44) | 7.32 (6.19) | <0.001 | <0.001 |
| Hypogonadism | 3 | 204.09 (65.33–637.58) | 202.04 (414.65) | 234.98 (75.78) | 7.88 (6.74) | <0.001 | <0.001 |
| Oral Candidiasis | 3 | 16.67 (5.35–52.01) | 16.52 (29.60) | 19.26 (6.21) | 4.27 (3.14) | <0.001 | 0.017 |
| Gamma-glutamyl transferase increased | 3 | 9.18 (2.94–28.63) | 9.10 (14.30) | 10.61 (3.42) | 3.41 (2.28) | 0.005 | 0.065 |
| Heavy menstrual bleeding | 3 | 7.69 (2.47–23.98) | 7.62 (11.29) | 8.89 (2.87) | 3.15 (2.02) | 0.008 | 0.093 |
| Iron deficiency anemia | 3 | 23.17 (7.43–72.28) | 22.95 (42.95) | 26.77 (8.63) | 4.74 (3.61) | <0.001 | 0.008 |

Note: *P* values were calculated using chi-square or Fisher’s exact tests based on 2×2 contingency tables. False discovery rate correction was performed using the Benjamini-Hochberg method, and q values represent adjusted p-values for multiple testing.

Abbreviations: PT, Preferred Term; ROR, reporting odds ratio; CI, confidence interval; PRR, proportional reporting ratio; χ^2^, chi-squared; EBGM, empirical Bayesian geometric mean; EBGM05, the lower limit of the 95% confidence interval for EBGM; IC, information component; IC025, the lower limit of the 95% confidence interval for IC.

**Supplemental Figure 1** Sensitivity analysis characterizing clinical and demographic features associated with serious outcomes excluding rare signals (N < 10). (A) Lollipop plot of standardized coefficients for the top 20 features derived from the elastic net logistic regression model, after excluding preferred terms with fewer than 10 report counts from the feature repository. The position and color of each dot represent the strength and direction of the multivariable association. (B) SHAP (SHapley Additive exPlanations) summary plot for the top 20 features derived from the Extreme Gradient Boosting (XGBoost) model under the same sensitivity framework. Each point represents an individual report, with its position on the x-axis quantifying the feature’s contribution to the serious outcome pattern.
